# Image Content Enhancement Through Salient Regions Segmentation for People With Color Vision Deficiencies

**DOI:** 10.1177/2041669519841073

**Published:** 2019-05-10

**Authors:** Alessandro Bruno, Francesco Gugliuzza, Edoardo Ardizzone, Calogero Carlo Giunta, Roberto Pirrone

**Affiliations:** INAF-IASF Palermo Istituto Nazionale di Astrofisica – Istituto di Astrofisica Spaziale e Fisica Cosmica, Palermo, Italy; Dipartimento dell’Innovazione Industriale e Digitale, Università degli Studi di Palermo, Palermo, Italy

**Keywords:** visual saliency, color vision deficiencies, image enhancement, eye-tracking, eye movements, image segmentation, imagery

## Abstract

Color vision deficiencies affect visual perception of colors and, more generally, color images. Several sciences such as genetics, biology, medicine, and computer vision are involved in studying and analyzing vision deficiencies. As we know from visual saliency findings, human visual system tends to fix some specific points and regions of the image in the first seconds of observation summing up the most important and meaningful parts of the scene. In this article, we provide some studies about human visual system behavior differences between normal and color vision-deficient visual systems. We eye-tracked the human fixations in first 3 seconds of observation of color images to build real fixation point maps. One of our contributions is to detect the main differences between the aforementioned human visual systems related to color vision deficiencies by analyzing real fixation maps among people with and without color vision deficiencies. Another contribution is to provide a method to enhance color regions of the image by using a detailed color mapping of the segmented salient regions of the given image. The segmentation is performed by using the difference between the original input image and the corresponding color blind altered image. A second eye-tracking of color blind people with the images enhanced by using recoloring of segmented salient regions reveals that the real fixation points are then more coherent (up to 10%) with the normal visual system. The eye-tracking data collected during our experiments are in a publicly available dataset called Eye-Tracking of Color Vision Deficiencies.

## Introduction

Scientific studies revealed that the most common form of color vision deficiency is encoded on the X sex chromosome; this is why color blindness is more widely diffused among males than females. Color vision deficiencies are mainly caused by protan, deutan, and tritan defects. Deutan color vision deficiencies are by far the most common forms of color blindness. This subtype of red–green color blindness affects about 8% of the male population, mostly in its mild form deuteranomaly (Simunovic, 2010). Red–green color blindness is split into two different types: Whereas people affected by protan color blindness are less sensitive to red light, deuteranopia or deuteranomaly (the second type of red–green color blindness) is related to sensitiveness on green light. Actually, color vision deficiencies include the following: protanopia, deuteranopia, tritanopia, protanomaly, deuteranomaly, and tritanomaly. The first three are types of dichromacy, which means only two different color receptors (cones) are in the retina instead of three (with normal color vision). The second three (protanomaly, deuteranomaly, and tritanomaly) go under the classification of anomalous trichromacy, which means all three different color receptors (cones) are present but one of them is shifted in its peak. Biological science focused on molecular genetics underlying color vision (Neitz & Neitz, 2011). Machado, Oliveira, and Fernandes (2009) simulated color vision by using a physiologically based model and handling normal color vision and color vision deficiencies such as anomalous trichromacy and dichromacy in a unified way. A lot of water passed under the bridge since Ishihara (1960) proposed the series of plates as test tool for color-blindness consisting of 38 isochromatic plates: The plates form an easy method for establishing the diagnosis and distinguishing cases of red–green deficiencies. The plates are held 75 cm from the subject and tilted so that the plane of the paper is at right angle to the line of vision. Since then, several models have been proposed as tool to detect color vision deficiencies. The Farnsworth–Munsell 100-Hue (FM100) test (Farnsworth, 1957) is a standardized measure of chromatic discrimination, based on colored cap-sorting, which has been widely used in both adults and children. During FM100 test, it is asked to order the shown color plates in the correct order, any misplacement can be related to a sort of color vision deficiency (Vingrys & Cole, 1983). RGB anomaloscope color blindness test consists of two different lamps with different lights to be matched, and it is a well-known and accurate tool to classify color blindness. It was developed by a German ophthalmologist more than 100 years ago, and it is still being used internationally to check color vision deficiencies and specific subtypes (Lakowski, 1969). A pseudoisochromatic color plate test called color vision testing made easy has been proposed by Cotter, Lee, and French (1999). It was designed for all age groups; it uses the identification of simple shapes and objects to detect red–green color deficiencies. Bimler, Kirkland, and Jameson (2004) quantified variations in color spaces with respect to sex differences. Gambino, Minafo, Pirrone, and Ardizzone (2016) presented a web application written in JavaScript that implemented a digital Ishihara-like test for preschool aged children. Y. S. Chen, Zhou, and Li (2016) delivered a color-blindness image (CBI) in order to deliver direct and effective information to dichromats by transforming CBIs into the pattern-highlighted image. Transform is made by means of color component analysis, pattern attention, and thresholding. The experiments confirmed the improvements of processing steps on CBI by means of Ishihara test plates.

Much of progress has been made in the last decades on simulating color vision-deficient systems (Brettel, Viénot, & Mollon, 1997; Kondo, 1990; Ichikawa et al., 2003, 2004; Meyer & Greenberg, 1988; Walraven & Alferdinck, 1997). Machado et al. (2009) proposed a method aimed at simulating the loss of chromatic contrast transforming the RGB image into an orthogonal dichromatic color space. Tajima and Komine (2015) developed a method based on visual saliency for quantifying and visualizing information loss and gain resulting from individual differences in spectral sensitivity. An algorithm that transforms color to grayscale preserving image detail by maintaining distance ratios during the reduction process is proposed by Rasche, Geist, and Westall (2005).

Some methods of the state of the art focused on the enhancement of colored regions from a visual attention perspective. The approach of Huang, Chen, Jen, and Wang (2009) is based on grouping the colors on Commission Internationale de l'Eclairage (CIE) L*a*b* space through a Gaussian Mixture model.

EyePilot (Perception Data Inc., 2006) is a fairly useful technique developed to assist color blind people in understanding and working with color-coded information. Jeong, Kim, Kim, Wang, and Ko (2012) proposed an image recoloring method based on color clustering with an information preserving property for color-blind people.

We focused our attention on how effective the enhancement of salient regions of an image is with dichromatic vision systems. We used visual saliency like a tool to detect the most important differences between normal and color vision-deficient systems.

Visual saliency (Ardizzone, Bruno, & Mazzola, 2011) deals with the identification of the most important regions of an image from a visual attention perspective; scientific studies reveal that human beings tend to observe the same regions of a visual scene (or an image) in the first seconds of observation. Eye-tracking the saccadic movements allows to extract a fixation point map giving us spatial information of the most observed locations of an image. Several scientific disciplines are involved in studying all the factors involved in visual attention such as psychology, medicine, biology, computer vision, and image processing.

The main objective of visual saliency is to imitate the behavior of the human visual system (HVS) during the first few seconds of observation by predicting where humans look. The output of a visual saliency system is a saliency map, that is, a two-dimensional grayscale image encoding the most salient regions of an image with values normalized to the range [0, 1]. Visual saliency approaches can be grouped as follows:

- Bottom-up approaches
- Top-down approaches
- Hybrid approaches.

Bottom-up approaches generally aim at detecting the most important regions of an image from a visual perception viewpoint by using low-level features as in literature (Achanta, Hemami, Estrada, & Susstrunk, 2009; Cheng, Mitra, Huang, Torr, & Hu, 2015; Harel, Koch, & Perona, 2007; Itti, Koch, & Niebur, 1998). Li et al. (2017) proposed a bottom-up approach for visual saliency detection in real time aiming at detecting and enhancing visual objects in the foreground of a scene. Top-down approaches detect visual saliency information by using high-level tools such as face, text, and object detectors (Kanan, Tong, Zhang, & Cottrell, 2009; Yang & Yang, 2017) Hybrid systems usually consist of the combination of bottom-up and top-down layers. In several hybrid approaches such as the Tsotsos and Rothenstein (Tsotsos, 2011) or Chen et al. (L. Q. Chen et al., 2003), top-down layer is used to refine the noisy map produced by bottom-up layer. Top-down layers are usually face detection or text detection modules or the combination of them. A well-known hybrid approach is the one proposed by Judd, Ehinger, Durand, and Torralba (2009a) in which low-, mid-, and high-level features have been used to train the saliency model (Judd, Ehinger, Durand, & Torralba, 2009b). Yu, Xia, Gao, and Samal (2016) work is based on Gestalt cues indices grouping, they focused their attention on objects saliency. Li and Yu (2016) presented a neural network for the purpose of saliency detection, they adopted convolutional neural network for detecting visual features at several scales. Toet (2011) made a comparison study to assess the performance of 13 saliency detection methods. Duncan and Sarkar (2012) provided some formal definitions of the three main saliency approaches, that is, bottom-up, top-down, and hybrid. Chavolla et al. (2018) performed a recoloring algorithm based on Hue, Saturation and Value (HSV) space color channels. Cercenelli et al. (2017) proposed a new MATLAB toolbox for saccade analysis to increase usability of eye-tracking systems in clinical ophthalmology practice. Korda, Asvestas, Matsopoulos, Ventouras, and Smyrnis (2015) classified three types of eye movements: saccades, blinks, and fixations by using pattern recognition techniques. Goldstein, Woods, and Peli (2007) recorded the eye movements of 20 normally sighted subjects as each watched six movie clips for the purpose of analyzing scanpath, eye-movement, and saccadics of people with visual impairments. In a recent work, Li et al. (2018) proposed a method on visual saliency segmentation for object recognition under simulated prosthetic vision. In our previous works (Ardizzone, Bruno, & Mazzola, 2013a, 2013b; Ardizzone, Bruno, & Gugliuzza, 2017), we proposed keypoint density maps as a tool to detect saliency from images. Since last decades, eye movements and computer science technologies have been really close for many tasks and related to biomedical applications and medical diagnosis (Freksa & Bertel, 2007; Han, Shin, Yoon, Jang, & Kim, 2014). In this article, we propose a new method for improving the color perception for people with color vision deficiencies such as protanopia, deuteranopia, protanomaly, and deuteranomaly. The main idea behind our work is that saliency maps can be used as crucial information to detect the most important differences between the images as perceived respectively by people with normal and deficient vision systems. We collected eye-tracking observations from people looking at color images with both normal and deficient vision systems. Eye-tracking data have been used both as ground truth and as maps to analyze and highlight the drawbacks of color vision-deficient systems with respect to the most important regions according to a normal vision system. To detect automatically the perceptual differences for a given image, we first make the image in a color blind version and then we extract the saliency maps of both the color and the color blind version of the image. The difference between the saliency maps gives us critical information about the regions to be segmented and recolored for the overall image enhancement for CVD people. Once the images have been enhanced, they have been used as test for eye-tracking experimental sessions with CVD people to assess the improvement from a perceptual viewpoint. The eye-tracking data we gathered during experimental sessions are made publicly available to be used as a ground-truth under the name of Eye-Tracking of Color Vision Deficiencies (EToCVD; Bruno, Gugliuzza, Ardizzone, Giunta, & Pirrone, 2018). The remainder of the article is organized as follows: In “Materials and Methods” section, we describe the steps of the method and the image datasets we used as test; in “Results and Discussion” section, we show our findings and discuss the relations between saliency-based segmentation and color blind image enhancement; and the “Conclusions” section ends the article.

## Materials and Methods

**Figure 1.**
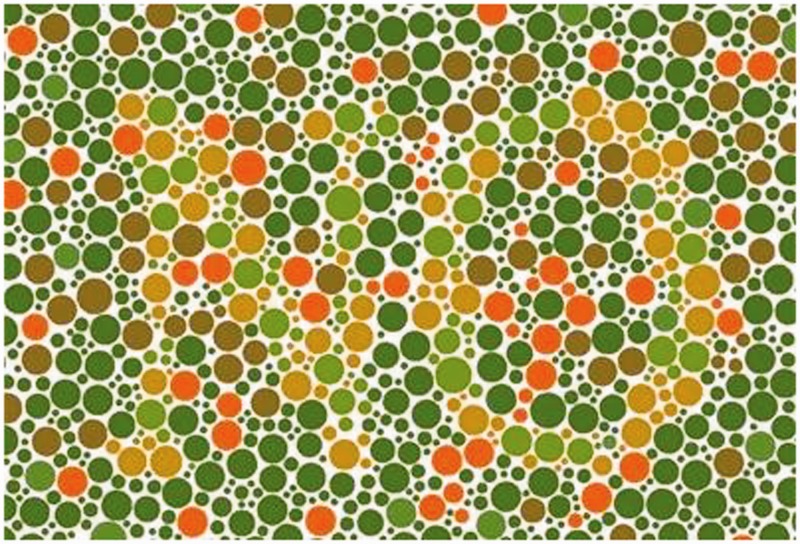
Unlike people with normal vision system, people with dichromatic vision system are able to easily recognize the word “NO.”

In this section, we describe the steps of our work, starting from the eye-tracking session, we aim at determining the most meaningful differences between normal and color vision-deficient systems with respect to a fixed number of images, then we tackle the segmentation and the recoloring of the regions with different saliency levels; at last, a further eye-tracking session assesses the enhancement of the images. We point out that we are interested in detecting differences in HVS behavior among people with normal vision system and people affected by color vision deficiencies (Figure 1 shows that only dichromatic people will be able to easily recognize the word NO standing out from the background).

### Eye-Tracking Session

The experimental sessions involved eight subjects with normal vision system and eight subjects with color-deficient vision system. More in detail, three subjects were affected by deuteranopia and five subjects were affected by protanopia. We conducted two experimental eye-tracking sessions: the first is focused on detecting how different are the fixation points among color blind and normal people, and the second is needed to assess the effectiveness of our method in enhancing the images for color blind people.

**Figure 2.**
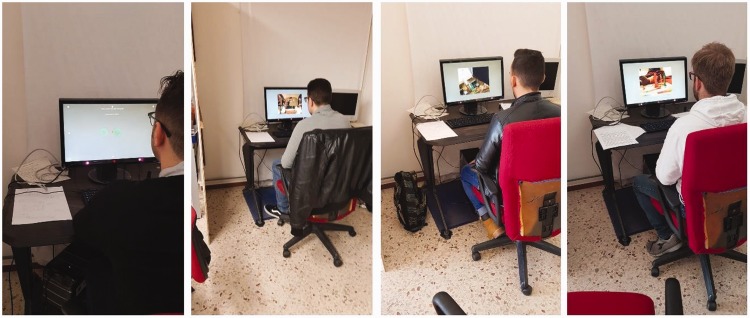
Images taken during the eye-tracking session: Starting from the calibration (the far-left image), the eye-tracker records the eye movements, the saccadic movements, and the scanpaths.

Both eye-tracking sessions consist of repeating the same procedures, but the first session also includes a test with Ishihara plates in advance to evaluate which kind of color vision deficiency the subjects (EnChroma Inc., 2010) are affected by. The experimental sessions have been conducted in a half-light room, and the subjects were kept at a distance of almost 70 cm from a 22-in. monitor with a spatial resolution of 1,920 × 1,080 pixel (Figure 2). During the eye-tracking session, a Tobii EyeX device recorded the eye movements, the saccadic movements, and the scanpaths of each subject while he or she was looking at the images shown on the screen. For each subject, a calibration step was needed to minimize saccadic movement tracking errors, to compute and assess the geometry of the setup (e.g., screen size, distance, etc.), and to collect measurements of light refractions and reflection properties of the corneas of each subject. Rather than using the standard Tobii EyeX Engine calibration (nine point calibration), we made use of Tobii MATLAB Toolbox 3.1 calibration whose procedure relies on a set of 13 points. Gibaldi, Vanegas, Bex, and Maiello (2017) added four targets in order to provide a finer coverage of the screen and a better evaluation of the residual calibration error by means of a greater spatial resolution. In our experimental sessions, each image was shown on the screen for a time of 3 seconds during which the Tobii EyeX acquired spatial coordinates of the eye movements (at a mean rate of 160 spatial coordinates per 3 seconds because of the sampling of about 55 Hz). Before switching to the next image, the screen was turned gray for 1 second to refresh the observer retina from the previous image signal. The eye-tracking session procedures follow the same overall scheme of previous scientific works (Ardizzone et al., 2011, 2013a, 2017) focused on visual saliency studies. Each session lasted approximately for 7 minutes per subject. For the purpose of our experiments, we created an ad hoc dataset by merging almost 90 images from different public datasets: MIT1003 (Judd et al., 2009a), CAT2000 (Borji & Itti, 2015), NUSEF (Ramanathan, Katti, Sebe, Kankanhalli, & Chua, 2010), MIT300 (Bylinskii et al., 2015): It consists of images containing meals, plants, objects, fruits, people, portraits, animals, pets, and synthetic pictures showing texture patterns. All the images have in common a prevalence of red–green chromatic contrasts (of interest for protanopia and deuteranopia deficiencies); we did not take into account images with yellow–blue chromatic contrast because we did not have available people affected by tritanopia vision deficiency. All the fixation point maps we collected during two experimental sessions have been gathered into a public available ground-truth under the name of EToCVD (Bruno et al., 2018).

**Figure 3.**
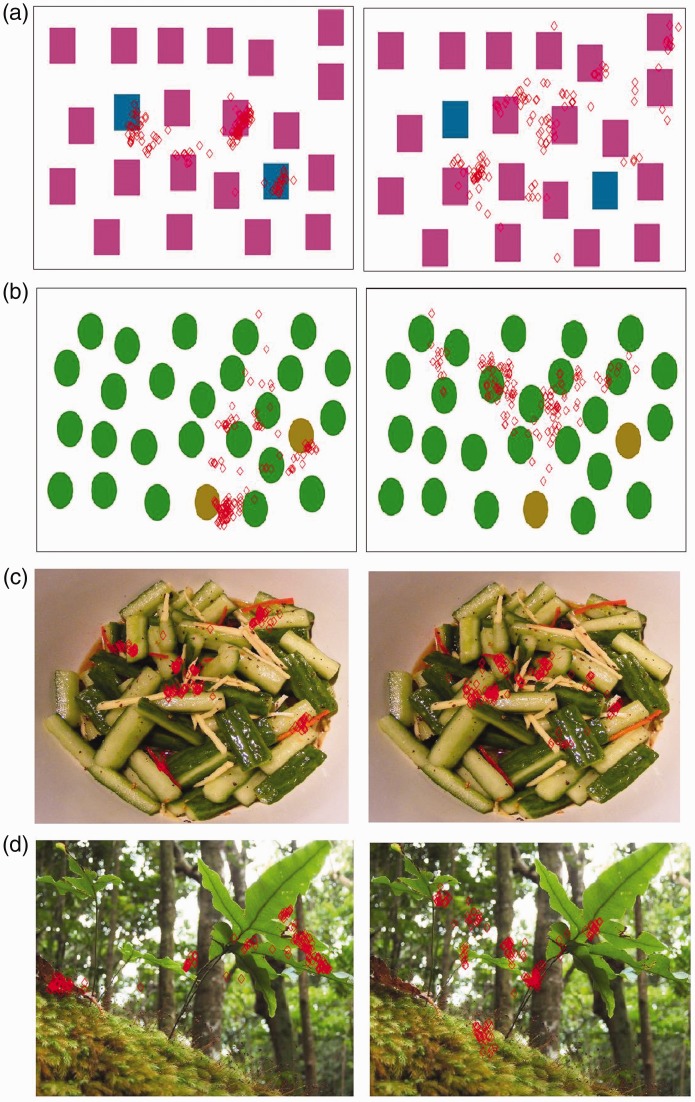
The visual perception of an image can be represented by the fixation points (red diamonds overlaid on the images) for both normal vision system (left column) and color blind vision system (right column). Some details are missed by people with color vision deficiencies and this is revealed by the lack of fixation points on the details noticed by people with normal vision system.

The eye movement data reveal the locations of the images looked by the observer during the experimental session. The fixation points are computed by averaging the spatial *x* and *y* coordinates of each eye movement (left and right eye movements). The coordinates are converted to the range of the spatial resolution of the screen. Each time a pixel is observed its value is incremented starting from zero. Then the saliency map is smoothed through a Gaussian convolution. Figure 3 shows how different the fixation points are between observers with protanopia and observers with normal color vision system. It is remarkable how people affected by protanopia miss several details because they fall within the color spectrum they are not able to discriminate. We want to point out that we aim to enhance the content of the image to make both people with deuteranopia and protanopia able to detect details they cannot detect because of the color blindness constraint.

### Image Enhancement Through Salient Regions Segmentation

For our purpose, we put our effort on assessing the usage of visual saliency in content enhancement for color blind people.

**Figure 4.**
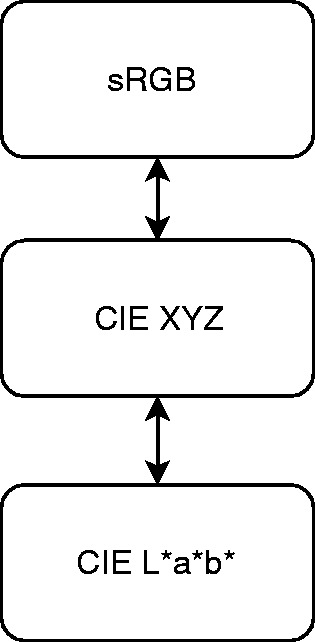
Due to the missing of a direct conversion between sRGB and CIE L*a*b*, first we went through a conversion between sRGB and CIE XYZ and between a CIE XYZ and CIE L*a*b* as shown in the scheme.

**Figure 5.**
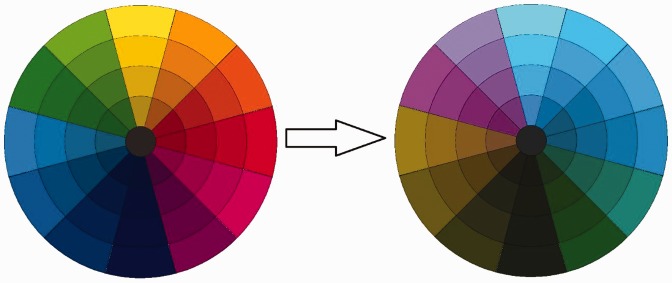
RGB to CIE L*a*b* conversion allows us to manage with color mapping within color frequencies well perceived by color blind people.

In our approach, for instance, the saliency map is extracted from an image by using the algorithm we proposed in our previous work (Ardizzone et al., 2017) based on the spatial distribution of local keypoints. Since we wanted to investigate the relation between the saliency and the color information, we tackled the saliency detection on CIE L*a*b* color space rather than sRGB (standard RGB) because of the independence of the luminance channel from color channels (a* and b*). A saliency map is computed along each channel of CIE L*a*b*, then the output saliency map is obtained by averaging the saliency maps of each channel. Providing that there is no direct conversion between sRGB and CIE L*a*b* color space, we went through a conversion between sRGB and CIE XYZ and then we applied a direct conversion between CIE XYZ and CIE L*a*b* as shown in Figure 4 giving rise to a color mapping like the one in Figure 5.

For a given sRGB system with (*x_r_*,*y_r_*), (*x_g_*,*y_g_*), and (*x_b_*,*y_b_*) as color space coordinates and (*X_W_*, *Y_W_*, and *Z_W_*) as white reference coordinates, we applied the conversions between sRGB and CIE XYZ color space as in transforms and Equations 1 to 3 as well described in the studies by Ford and Roberts (1998) and Ohta and Robertson (2006).

(1) $$\begin{array}{c} \begin{array}{c} {}[\begin{array}{c} X\\ Y\\ Z \end{array}]=[\begin{array}{c} M \end{array}][\begin{array}{c} R\\ G\\ B \end{array}] \end{array} \end{array}$$

where

(2) $$\begin{array}{c} \begin{array}{c} {}[\begin{array}{c} M \end{array}]=[\begin{array}{ccc} S_{r}X_{r} & S_{g}X_{g} & S_{b}X_{b}\\ S_{r}Y_{r} & S_{g}Y_{g} & S_{b}Y_{b}\\ S_{r}Z_{r} & S_{g}Z_{g} & S_{b}Z_{b} \end{array}] \end{array} \end{array}$$

where

(3) $$\begin{array}{cc} X_{r} & =x_{r}/y_{r};\\ Y_{r} & =1;\\ Z_{r} & =(1-x_{r}-y_{r})/y_{r};\\ X_{g} & =x_{g}/y_{g};\\ Y_{g} & =1;\\ Z_{g} & =(1-x_{g}-y_{g})/y_{g};\\ X_{b} & =x_{b}/y_{b};\\ Z_{b} & =(1-x_{b}-y_{b})/y_{b} \end{array}$$

Once the conversion between RGB and CIE XYZ was accomplished, we managed to convert the image between CIE XYZ and CIE L*a*b* as described in Equations 4 to 8.

(4) $$\begin{array}{c} \begin{array}{c} L^{*}=116f_{y}-16\\ a^{*}=500(f_{x}-f_{y})\\ b^{*}=200(f_{x}-f_{z}) \end{array} \end{array}$$

where

(5) $$\begin{array}{c} \begin{array}{c} f_{x}=\{\begin{array}{cc} \sqrt[{3}]{x_{r}}, & \text{ if }\;x_{r}>\in \\ \frac{{\mathrm{kx}}_{r}+16}{116}, & \text{ otherwise } \end{array} \end{array} \end{array}$$

(6) $$\begin{array}{c} \begin{array}{cc} f_{y} & =\{\begin{array}{cc} \sqrt[{3}]{y_{r}}, & \text{ if }\;y_{r}>\in \\ \frac{{\mathrm{ky}}_{r}+16}{116}, & \text{ otherwise } \end{array} \end{array} \end{array}$$

(7) $$\begin{array}{c} \begin{array}{c} f_{z}=\{\begin{array}{cc} \sqrt[{3}]{z_{r}}, & \text{ if }\;z_{r}>\in \\ \frac{{\mathrm{kz}}_{r}+16}{116}, & \text{ otherwise } \end{array} \end{array} \end{array}$$

(8) $$\begin{array}{c} \begin{array}{c} x_{r}=\frac{X}{X_{r}}\\ y_{r}=\frac{Y}{Y_{r}}\\ z_{r}=\frac{Z}{Z_{r}} \end{array} \end{array}$$

**Figure 6.**
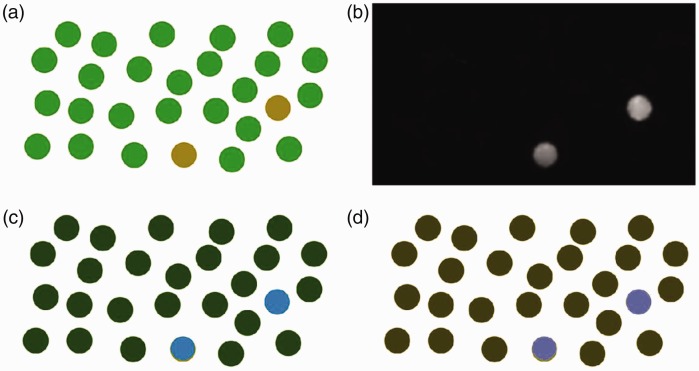
(b) The saliency error is computed as the difference of the saliency maps of (a) the original image and the color blind version of the image. (c) The saliency error regions are segmented and color boosted in CIE L*a*b* color space by using the opposite value with the a* and b* channels, and (d) the enhancement is also mapped in the color blind domain.

**Figure 7.**
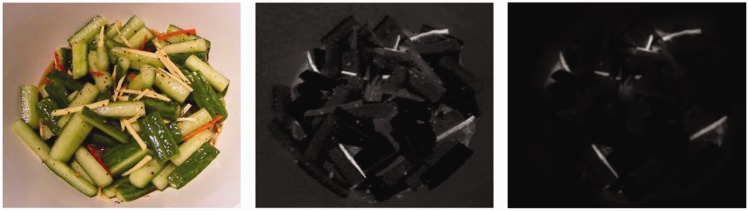
The highlights of perceptual differences. For a given image (left), some enhancement methods use the average of the differences of L*a*b* channels between the original image and the color blinded version (center). We adopted the difference of L*a*b* channels between original image and the version with color blind weighted by the saliency difference (right).

Parameters such as ∈ and *k* in the aforementioned equations are defined by CIE standard (Ohta & Robertson, 2006), *X_r_*, *Y_r_*, and *Z_r_* represent the white reference coordinates with respect to red, blue, and green components. Given the image in the CIE L*a*b* color space, a color vision deficiency simulation method (Milić, Hoffmann, Tómács, Novaković, & Milosavljević, 2015), in the same way of Viénot, Brettel, and Mollon (1999), is applied to have a dichromatic version of the original image. Vienot’s method allows us to choose the color deficiency to be simulated through a function parameter. Afterward, the saliency map of the simulated dichromatic version of the image is extracted by using the same procedure as shown in the study by Ardizzone et al. (2017). The saliency error is used as a weight and multiplied with the difference between the original image and the simulated dichromatic version (see Figure 7), then the result is converted from CIE L*a*b* to RGB space by using the inverse of transforms and Equations 1 to 8. A correction vector is multiplied with the resulting RGB image and an average function is applied along the three RGB channels giving rise to a single map, furthermore a 3 × 3 sized Gaussian filter is applied to smooth the map noise. The smoothed map is segmented by using the adaptive Otsu (1979) segmentation. The saliency error is then represented as segmented regions. For the enhancement purpose only, the segmented regions have been taken into account (see Figure 6), that is, the pixels of the segmented regions are transformed with a negative mapping as in the equations reported in Equation 9 that represent a rotation of 180° of the a* and b* channels in CIE L*a*b* space:

(9) $$\begin{array}{c} \begin{array}{c} L^{*'}=L^{*}\\ a^{*'}=-a^{*}\\ b^{*'}=-b^{*} \end{array} \end{array}$$

Using the aforementioned equations and then converting the resulting image back to RGB color space, we noticed that at first sight, color blind people were able to perceive more details from the regions with pixels falling within the color frequency that they were not able to discriminate before. More in detail, as can be seen in Figure 5 and the equations reported earlier, hues close to red are mapped to hues very close to blue spectrum. Before the aforementioned processing steps, people affected by protanopia and deuteranopia were not able to discriminate red edges over green background. A new eye-tracking session has been conducted to assess the effectiveness of the aforementioned enhancement and processing steps with the visual perception of color blind people.

## Results and Discussion

**Figure 8.**
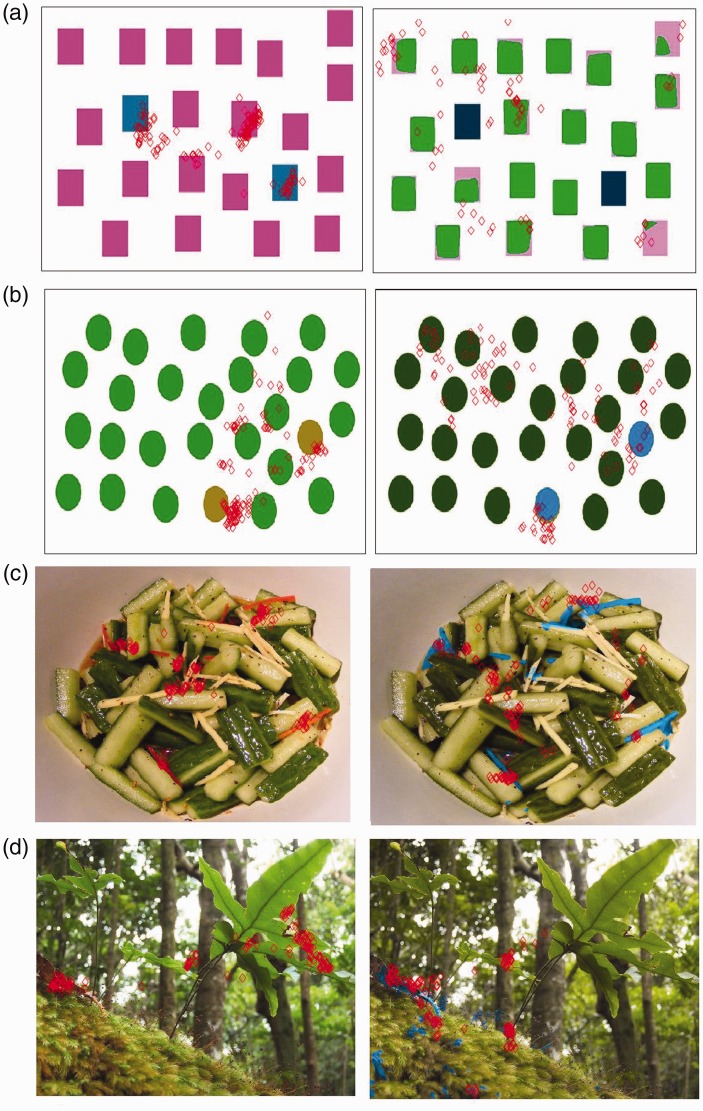
The fixation points (red diamonds overlaid on the images) of observers with normal color vision system (left column) and the ones of people with protanopia. The images from the right column are from the second eye-tracking session when observing the images enhanced by our method.

In this section, we want to describe our findings with respect to the behavior of people with protanopia and deuteranopia, after that we performed the enhancement processing steps highlighted in the previous section. Once the images had been enhanced, we repeated the eye-tracking session (20 days after the first one) only with subjects affected by color vision deficiencies. The idea behind a second experimental session is to assess the effectiveness of our enhancement approach by comparing the real fixation points directly on the same images. We considered the real fixation point maps of the observers with normal color vision system as our ground truth. First, we measured the distance of color blind people real fixation point maps with respect to our ground truth after the first eye-tracking experimental session and we used that as reference for our comparison studies. Then, we computed the distance between real fixation point maps related to the second eye-tracking session and the ground truth. We conducted experiments with people affected by protanopia and deuteranopia and we collected the real fixation point maps to be evaluated with metrics such as normalized scanpath saliency (NSS) and area under curve (AUC) focused on visual perception processes. Very interesting results can be observed from the images in Figure 8. The observers affected by protanopia were able to discriminate and notice some details they did not look at before (Figure 5). Despite some imperfections in the recoloration of the segmented region (Figure 8(a)), we noticed that the overall distribution of the fixation points (Figure 8(b), (c), and (d)) over the images is closer to the corresponding ground truth map than the distribution of the fixation points obtained during the first eye-tracking session. As shown in Figure 11(a), (b), (c), and (d), the eye movements of the observers affected by protanopia and deuteranopia can be really different than those of people with normal color vision system. The interesting thing is that, analyzing the improvements obtained with our enhancement method by observing the fixation points map of subjects affected by protanopia (Figure 12(c)) and deuteranopia (Figure 12(d)), the improvement is noticeable because the fixation points are quite closer to our ground truth (Figure 11(a)).

The performance of our method is depicted by using metrics such as AUC and NSS well suited for quantifying how close are fixation point maps of color blind people to the fixation point maps of people with normal vision system. For the sake of clarity, we studied the performance of our method on people with protanopia and deuteranopia separately.

We want to point out that scientific literature on visual attention revealed that during the first 200 milliseconds of an image observation, humans tend to fix locations around the image center, and this resulted in a center-biased fixation point map. It is of our interest to analyze the experimental results by distinguishing two case studies:

- Collecting fixation point data of the observer over the entire time interval for each image (3 seconds)
- Collecting fixation point data from 200 milliseconds to 3 seconds.

The objective of excluding the first 200 milliseconds from the fixation point data is to have unbiased data to be analyzed.

**Figure 9.**
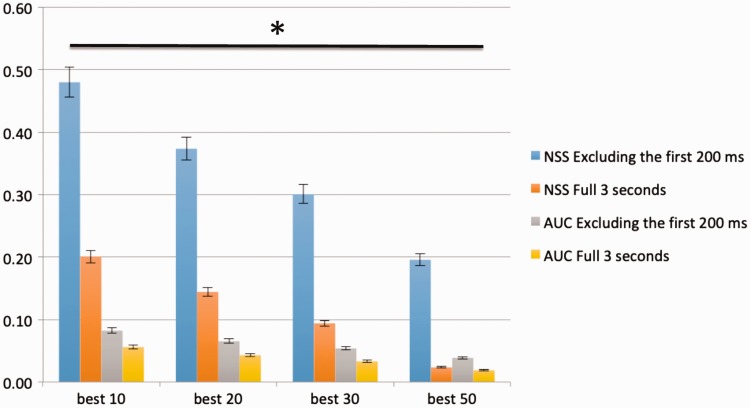
Average NSS and AUC score of the best 10, 20, 30, and 50 cases within protanopia case study. Repeated-measures ANOVA returned *p between groups lower than .05.

**Figure 10.**
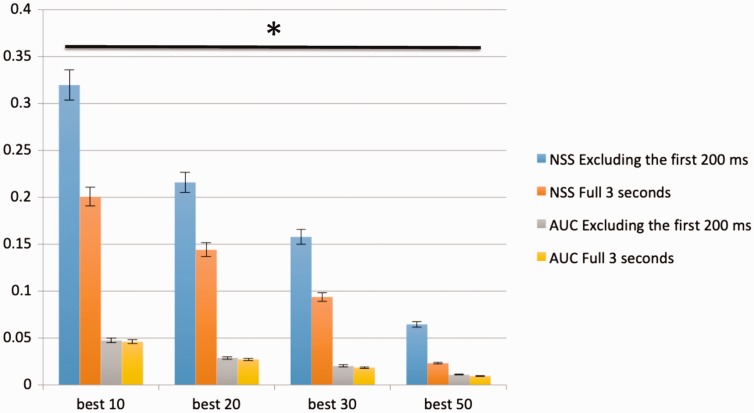
Average NSS and AUC score of the best 10, 20, 30, and 50 cases within deuteranopia case study. Repeated-measures ANOVA returned *p between groups lower than .05.

NSS allows us to give a measure of how close a saliency map is to a real fixation point map. The metric was originally thought to compute the distance between a computational saliency map and a real fixation point map. In our method, we used NSS to compute the distance between the real fixation point map of people with normal vision system and the real fixation point map of people with color vision deficiencies. NSS metric gives us a scalar value. An NSS value of zero means the maps are very different, conversely, a higher NSS value means higher similarity between the maps. AUC metric is computed as the area of the receiver operator characteristic curve. It is a scalar representation of the predicted performances of a classifier. AUC value falls within the range [0, 1]. Looking at the AUC and NSS results of the protanopia case study (Figure 9), we noticed that the enhancement of the images allowed observers to detect more details previously falling in the color blind spectrum. Our results reached an average score increase of approximately 0.08 AUC and 0.5 NSS (both excluding the first 200 milliseconds). Figure 13 shows some examples with different AUC and NSS values related to quite meaningful images with the corresponding eye-tracking fixation point map of the first eye-tracking session and the fixation point map related to the second eye-tracking session, giving us a visual and qualitative demonstration of the improvement we achieved. In Figure 10, we plotted the histogram graph of AUC and NSS average score increase with respect to the deuteranopia case study and we showed meaningful images and the corresponding fixation point maps. It is noticeable that in the case of observers with protanopia, we reached an average score increase of approximately 0.05 AUC and 0.3 NSS (both excluding the first 200 milliseconds). As you can see from the histogram bars, there are a lot of differences between including and excluding the first 200 milliseconds in our case study. It is evident that observers with protanopia show a more peculiar center bias in their visual attention path. We applied a color mapping function who takes into account both deuteranopia and protanopia effect, and we looked for a trade-off mapping function allowing to achieve the best improving results for both kind of color vision deficiencies. So far, results showed a better improvement for people affected by deuteranopia (in Figure 14 you can see some experimental results with respect to deuteranopia case study); this may be explained by referring to the effectiveness of the negative color mapping as in Equation 9 that is more appropriated with respect to deuteranopia than protanopia. We will be focusing on two different mapping functions to be tuned on the two color deficiencies differently. Besides the results related to color blind people visual perception, we want to show the running performance of the image enhancement method as described in Section “Image Enhancement Through Salient Regions Segmentation.” As shown in Table 1, we do not have an average running time but an average range depending on the saliency map extraction. The reason behind is that the saliency method we adopted as saliency map extraction is mainly based on the number of scale invariant feature transform (SIFT) keypoint (Lowe, 1999) detected in the image which in turn depends on the size of the image and on the textured regions in the image (the finer the texture in the image, the greater the number of scale-invariant feature transform keypoints we have in the image). The experiments have been conducted by using a Tobii EyeX eye-tracker recording the eye movements with a sampling rate of about 55 Hz; the data have been processed in MathWorks MATLAB, in greater detail we dealt with Tobii EyeX calibration and eye-tracking parameter tuning inside TobiiMatlabToolbox 3.1 (Gibaldi et al., 2017; Gibaldi, Vanegas, Bex, & Maiello, 2018). We used a workstation with a quad-core 2.4 GHz processor and 16 GB of RAM for our experiments.

**Figure 11.**
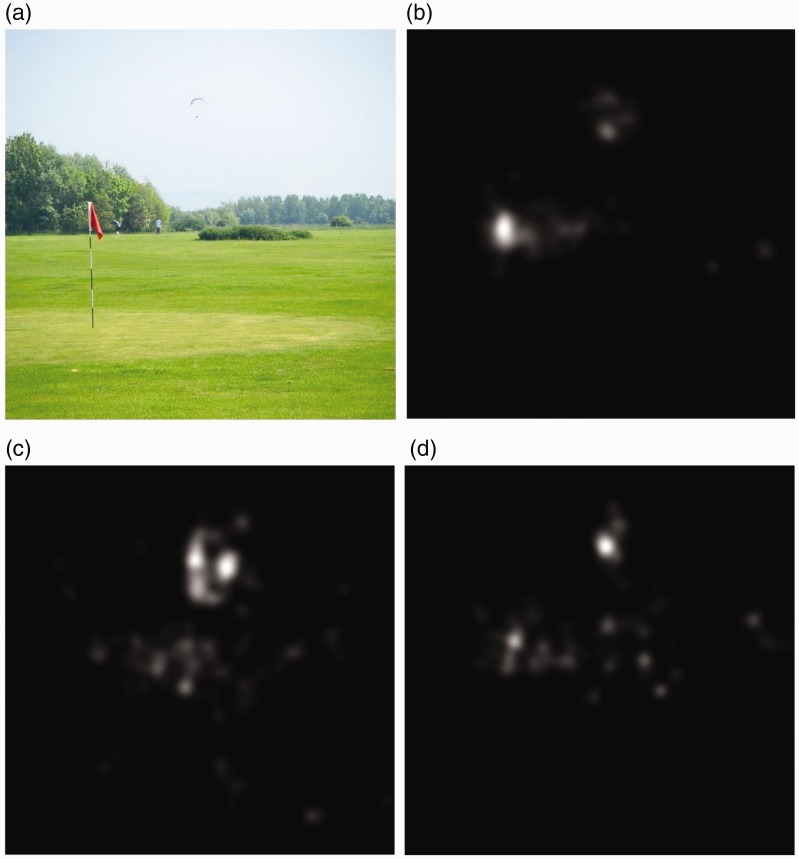
For (a) a given image we collected the fixation points from (b) a normal observer, (c) an observer with protanopia and (d) an observer with deuteranopia.

**Figure 12.**
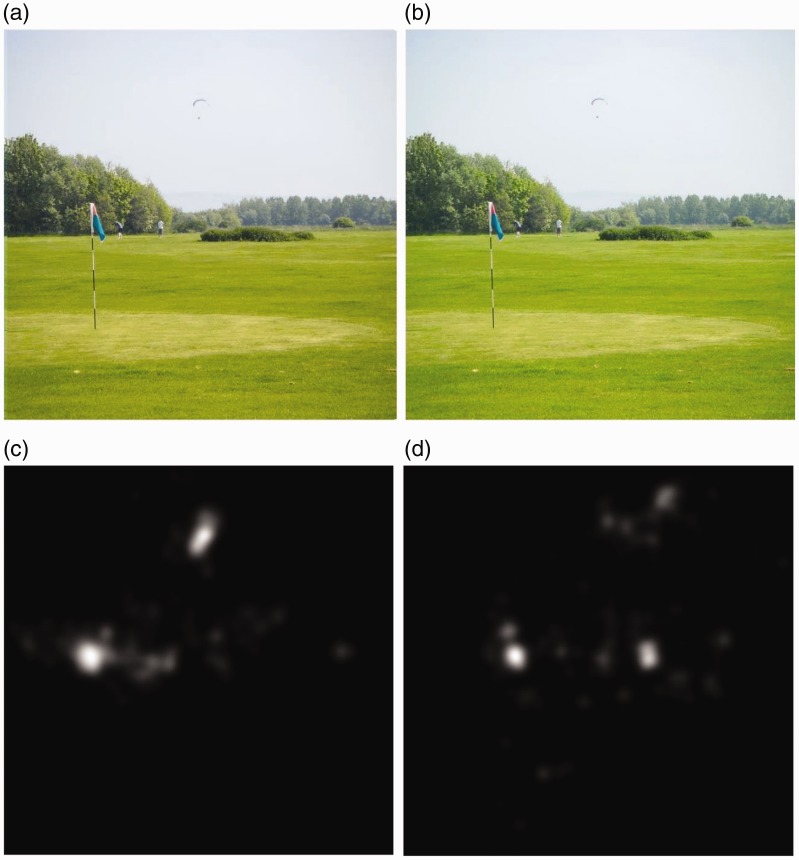
The enhancement assessment on (a and b) the images is supported by the fixation point maps for observers with (c) protanopia and (d) deuteranopia.

**Figure 13.**
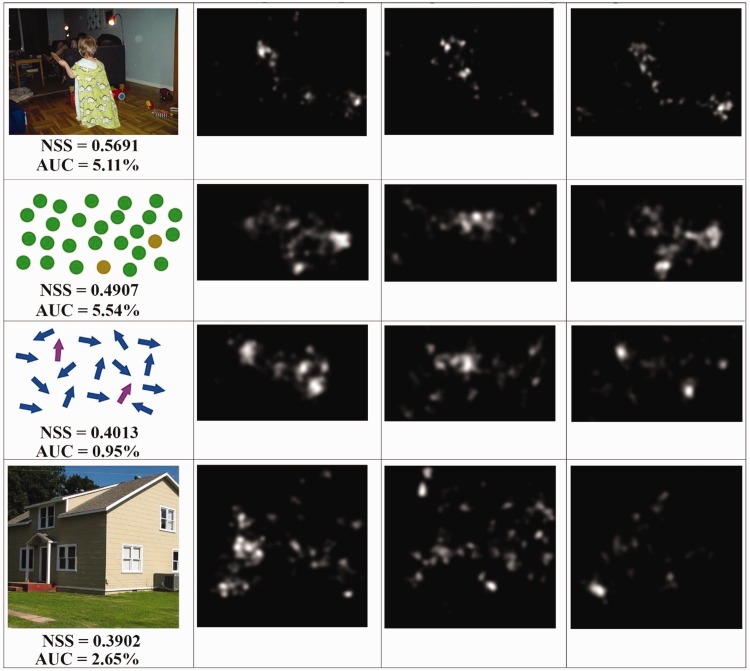
For a given image (first column), we collected the fixation points from normal observer (second column), from observers with protanopia (third column), from observers with protanopia looking at the enhanced image (fourth column).

**Figure 14.**
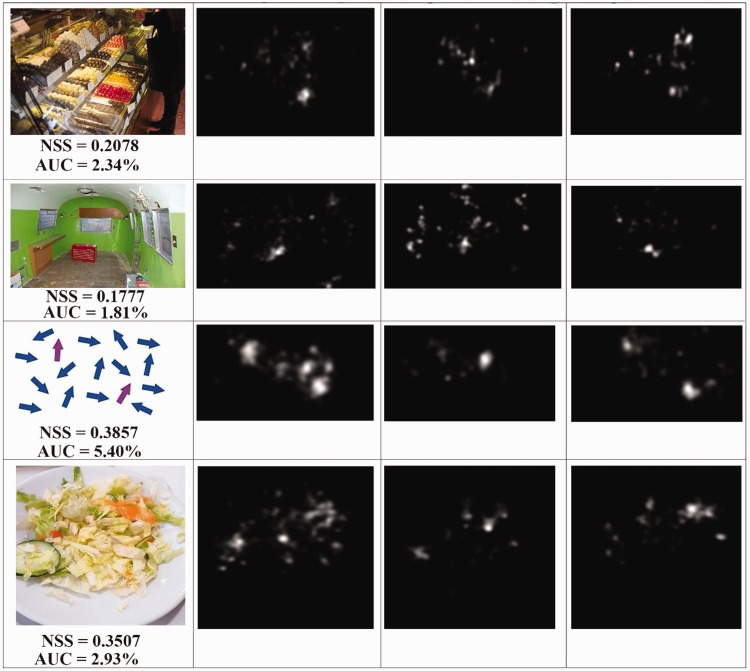
For a given image (first column), we collected the fixation points from normal observers (second column), from observers with deuteranopia (third column), from observers with deuteranopia looking at the enhanced image (fourth column).

**Table 1.** From Left to Right We Show the Running Time Ranges of Both the Overall Image Enhancement (Applied as Described in Section “Image Enhancement through Salient Regions Segmentation”) and Its Most Important Steps: Color Vision Deficiency Simulation, Saliency Map, Map Segmentation, and a Negative Color Mapping as in Equation 9 in CIE L*a*b* Space.

Overall image enhancement	CVD simulation	Saliency map	Map segmentation	Color mapping
4–75 s	0.6–5.6 s	2.9–61.1 s	5.3e-4–0.5e-1 s	0.73–5.7 s

*Note.* CVD = color vision deficiencies; CIE = Commission Internationale de l'Eclairage.

## Conclusion

The HVS tends to fix some specific points and regions of the image in the first few seconds of observation summing up the most important and meaningful parts of the scene. In this article, our findings are related to the differences of eye movements with respect to normal and color vision-deficient visual systems. Two eye-tracking experimental sessions allowed us to detect and analyze the image details that are not well perceived and fixed by color blind observers. We provided a method to enhance color regions of the image based on CIE L*a*b* color mapping of segmented salient regions. The segmentation is performed by using a saliency-weighted difference between the original input image and the corresponding color blind altered image. A second eye-tracking session with color blind people on the enhanced images revealed that the real fixation points are then more coherent with the normal visual system: up to 10% for people with protanopia and up to 5% for people with deuteranopia. The method we proposed makes color blind people able to detect some more red–green details from the images with respect to the original image. We are now working to improve the method solution for protanopia color deficiency, by investigating the entire spectrum on CIE L*a*b* color space. We need to find a more specific color mapping to deal with protanopia. We also want to optimize our code and develop a lightweight version that might be installed on wearable devices (glasses at first) aiming to assess how comfortable and ecological it could be having an enhanced visual experience in everyday life. We also provided a new public dataset under the name of EToCVD (Bruno et al., 2018) gathering the real fixation point maps for both normal and CVD affected people involved during our experimental sessions.
